# Is day-case surgery feasible for laser endoscopic enucleation of the prostate? A systematic review

**DOI:** 10.1007/s00345-023-04594-7

**Published:** 2023-09-10

**Authors:** Mehmet Yilmaz, Mustafa Karaaslan, Muhammed Emin Polat, Senol Tonyali, Halil Çağrı Aybal, Mehmet Emin Şirin, Tuncay Toprak, Lütfi Tunç, Christian Gratzke, Arkadiusz Miernik

**Affiliations:** 1 Department of Urology, Medical Centre - University of Freiburg, Faculty of Medicine, Hugstetter Str. 55, 79106 Freiburg, Germany; 2 Clinic of Urology, Bingol State Hospital, Bingol, Turkey; 3 Department of Urology, University of Health Sciences, Ankara City Hospital, Ankara, Turkey; 4 Department of Urology, Istanbul University Istanbul School of Medicine, Istanbul, Turkey; 5 Clinic of Urology, Polatlı Duatepe State Hospital, Ankara, Turkey; 6 Department of Urology, University of Health Sciences, Diskapi Yildirim Beyazit Training and Research Hospital, Ankara, Turkey; 7 Department of Urology, University of Health Sciences, Fatih Sultan Mehmet Training and Research Hospital, Istanbul, Turkey; 8 Faculty of Medicine, Department of Urology, Gazi University, Ankara, Turkey

**Keywords:** Benign prostatic hyperplasia, Day-case surgery, Laser endoscopic enucleation of the prostate, Same day discharge

## Abstract

**Purpose:**

Laser endoscopic enucleation of the prostate (EEP) for benign prostatic obstruction has become increasingly prevalent worldwide. Considering the medical cost-savings and concomitantly fewer nosocomial infections, the feasibility of same-day postoperative discharge of patients who have undergone laser EEP in terms of its safety and effectiveness has become a subject matter of growing interest. We aimed to review those studies focussing on day-case surgery (DCS) in patients undergoing laser EEP.

**Methods:**

A systematic search was conducted using PubMed-MEDLINE and Web of Science databases until October 2022 with the following search terms: “same day discharge AND laser enucleation of the prostate”, “day-case AND laser enucleation of the prostate”, “same day surgery AND laser enucleation of the prostate” and “one day surgery AND laser enucleation of the prostate” by combining PICO (population, intervention, comparison, outcome) terms. We identified 15 eligible studies.

**Results:**

While 14 of the studies focussed on holmium laser EEP, one focused on thulium laser vapoenucleation of the prostate. We observed an improvement in functional parameters in all studies we reviewed, and DCS success and readmission rates ranged between 35.3–100% and 0–17.8%, respectively. The complication rates varied between 0 and 36.7%, most of the complicatons were Clavien-Dindo (CD) I and II. CD ≥ III complications did not significantly differ between same day discharge (SDD) and non-SDD groups in the studies.

**Conclusion:**

Laser EEP is feasible and promising DCS treatment option delivering improved functional parameters compared to baseline values, and lower perioperative complication and readmission rates in certain patients.

**Supplementary Information:**

The online version contains supplementary material available at 10.1007/s00345-023-04594-7.

## Introduction

Benign Prostatic Enlargement (BPE)-related lower urinary tract symptoms (LUTS) are an important adult male health problem that worsens with age, causing personal and social distress [1]. Laser endoscopic enucleation of the prostate (EEP) in the surgical treatment of BPE has become much more prevalent since Gilling et al. first described it in 1998 [2]. Laser EEP has been proven to be an effective alternative BPE intervention to Transurethral Resection of the Prostate (TURP) and Open Prostatectomy (OP) in terms of its efficacy, safety and perioperative complications, as it is doable regardless of prostate size [3–8]. Laser EEP is also associated with low morbidity, i.e. a lower risk of intraoperative bleeding and blood transfusions [9, 10].

Laser EEP is comparable to traditional transurethral and OP procedures in its functional results, but it is superior to them because shorter hospital stays naturally result in fewer perioperative complications [11]. Considering the factors of medical expenses and nosocomial infections, keeping the postoperative hospital stay short is an obvious benefit. Therefore, the feasibility as well as the safety and effectiveness of same-day discharge of patients undergoing laser EEP has been a subject of growing interest recently, attracting considerable research attention [12–18]. However, there has been no study to date evaluating these investigations from the same-day discharge perspective after laser EEP offering a comprehensive conclusion. We therefore aimed to review those studies addressing the same-day discharge of laser EEP patients and thereby discuss the feasibility and safety of day-case laser EEP surgery.

## Materials and methods

### Search strategy

This study was conducted in compliance with the Preferred Reporting Items for Systematic Reviews and Meta-analyses (PRISMA) statement [19]. A systematic search was conducted using PubMed-MEDLINE and Web of Science databases until October 2022 with the following search terms: “same day discharge AND laser enucleation of the prostate”, “day-case AND laser enucleation of the prostate”, “same day surgery AND laser enucleation of the prostate” and “one day surgery AND laser enucleation of the prostate”. After retrieving the titles and abstracts of selected articles, the full texts of related articles were screened. The reference lists in all relevant articles and reviews were also checked.

The objective of this systematic review was to assess the feasibility and safety of laser EEP as a same-day surgery in patients suffering symptomatic BPE according to functional parameters such as International Prostate Symptom Score (IPSS) or American Urological Association Symptom Score (AUASS), maximum urinary flow rate (Qmax), post-void residual (PVR), perioperative complications according to Clavien–Dindo (CD) classification, hospital readmission rates and readmission reasons.

### Eligibility criteria

Our search process is shown in PRISMA flow diagram (Online Resource 1—Fig. 1). As recommended in the PRISMA guidelines, the PICO, Population (P), Intervention (I), Comparison (C), Outcomes (O), approach was taken to assess eligibility criteria [20]. We thus selected studies comparing BPE patients (P) who underwent laser EEP (I) including those discharged on the same day after the operation (C) to assess postoperative functional outcomes, perioperative complications and safety (O). Excluded were any studies not associated with laser enucleation of the prostate, or not addressing objectives or outcomes related to mainly same-day discharge regarding surgical outcomes, or review articles, as well as case reports, articles not written in English, conference abstracts, editorials/letters or reply to authors.

### Data extraction

Articles relevant to our subject of interest were retrieved and evaluated independently by two authors (M.K. and M.E.P.) and discrepancies were resolved through discussion by a third reviewer (T.T.). We documented authors and date of study, study design, patient numbers, age, preoperative prostate volume (PV), preoperative total serum PSA (ng/ml), preoperative and postoperative IPSS or AUASS, Qmax (ml/s), PVR (ml), quality of life (QoL), previous prostate surgery, usage of 5α*-*Reductase inhibitors (5-ARI) and antiplatelet/anticoagulation (AP/AC), operative time (OT) (min), enucleated prostate weight, enucleation (ET) and morcellation times (MT) (min), enucleation efficiency (EE), morcellation efficiency (ME) (g/min), duration of catheterisation (DOC) (days), length of hospital stay (LOS), postoperative readmission rate, the reason for readmission and management of readmissions, success rate of same-day discharge and perioperative complications according to the Clavien–Dindo classification. Descriptive statistics were used to present basic data.

### Quality assessment of the studies

We examined and assessed the quality of evidence in the studies using the National Institutes of Health Quality (NIH) Assessment Tool for Pre-Post Studies without Control Groups. (https://www.nhlbi.nih.gov/health-topics/study-quality-assessment-tools). This tool consists of 12 questions assessing study quality with the options "yes", "no", "cannot be determined", "not applicable" or "not reported" for each question, respectively. Discrepancies between two authors were resolved through discussion. Quality ratings were indicated according to other reviews: poor (< 60%), adequate/fair (60–69%), good (70–79%) and strong (80%) [21, 22]. A quality percentage score for each study was determined by dividing the number of “yes” answers by the total number of valid questions. 

## Results

We identified a total of 167 studies via our search of the databases. Of those, 148 were excluded after the title and abstract evaluation for the following reasons: duplicate articles (*n* = 71), inclusion criteria not met or unrelated to laser enucleation of prostate, objective or outcome not associated with mainly same-day discharge (*n* = 51), review articles (*n* = 8), case reports (*n* = 3), editorials/letters (*n* = 2), conference abstracts (*n* = 4) and not written in English (*n* = 9). After full-text evaluation, we excluded another four articles that failed to address day-case surgery for laser EEP.

Five studies were conducted prospectively [12, 15–17, 23], while ten were retrospective [13, 14, 18, 24–30]. While 14 studies focussed on Holmium Laser Enucleation of the Prostate (HoLEP) [12–14, 16–18, 23–30], one was on thulium laser vapoenucleation of the prostate (ThuVEP) [15]. Six of the studies compared the findings of patients discharged on the same day of surgery to patients not discharged on the same day [13, 16, 18, 25, 26, 30]. According to NIH Quality Assessment Tool, 6 studies were found to be of “poor”, 3 to be of “adequate/fair”, 2 studies were judged to be of “good” and 4 studies were judged to have “high” quality (Online Resource 1—Table 1).

Patient inclusion and exclusion criteria for each study are given in Table 2 (Online Resource 1).

### Perioperative and urinary outcomes

Table 3 (Online Resource 2) presents the summary of baseline characteristics and perioperative data from the studies included in the present review. Table 4 (Online Resource 3) illustrates the patients’ intraoperative and postoperative data.

In their study including 30 patients, Agarwal et al. discharged the patients on the same day as HoLEP surgery and evaluated same-day removal of the urethral catheter [24]. They observed an improvement in postoperative AUASS [18 (IQR 13–29) vs. 5 (IQR 2–5)]. In their retrospective study with 55 patients, Assmus et al. reported same-day discharge outcomes of patients presenting prostate glands > 175 cc in size who underwent HoLEP [14]; their same-day discharge success rate was 84%, and they reported an improvement in all patients’ AUASS and Qmax values at the postoperative third month of follow-up (22.3 vs 6.7; *p* < 0.001, 8.8 vs 20.4 ml/sec; *p* < 0.001).

Larner et al. performed HoLEP surgery in 38 patients with a prostate size < 60 ml, discharging their patients on the same day with an indwelling catheter [27]. Their IPSS and Qmax values at the postoperative third month improved compared to baseline values (23.8 ± 7.3 vs. 6.6 ± 6.8 for IPSS and 6 ± 3.3 vs. 18.4 ± 8 for Qmax). Comat et al. planned to discharge 90 of 211 patients who underwent HoLEP on the same day of surgery; most of their patients (83.4%) were discharged within 12 h postoperatively [23]. They also reported improved postoperative IPSS and Qmax values (20 vs. 5.04 for IPSS and 7.4 vs. 24.9 mL/s for Qmax).

Lee et al. carried out HoLEP in 210 patients, discharging 74 patients on the same day of surgery with a 35.3% success rate [29]. However, they did not assess preoperative and postoperative functional parameters. In another retrospective study, Lee et al. compared Moses 2.0-augmented HoLEP (*n* = 192) (m-HOLEP – a new laser technology employing pulse modulation making laser energy delivered to the target more efficient) to standard HoLEP (*n* = 120) in terms of cost, same-day discharge and emergency department admission/readmission data [28]. They suggest that m-HoLEP enables better haemostasis and is suitable for day surgery as enucleation takes less time. Perioperative functional parameters were not evaluated in that study. The average hospital expenses, encompassing equipment use and expendable materials were observed to be significantly lower for m-HoLEP in comparison to HoLEP (*p* = 0.0297). Upon examination of total expenses, inclusive of surgical cost and postoperative expenditures within a 30-day period (including emergency department visits and hospital readmissions), m-HoLEP demonstrated a cost reduction of $747 per case for the hospital, although this finding was close to the threshold of statistical significance (*p* = 0.0574). The authors linked these cost savings to two primary factors: same-day discharge and shorter duration of surgery.

In their prospective study, Abdul-Muhsin et al. scheduled 47 patients for same-day discharge among a group of 179 patients who underwent HoLEP [12]. Of that group, 28 (59.5%) patients were successfully sent home on the same day of surgery. They did not evaluate perioperative functional outcomes in their entire cohort; exclusion criteria were age > 75 years, prostate size > 200 cc and ASA > 3. In their retrospective study with one of the largest day-case HoLEP cohorts in the literature, Klein et al. discharged 214 of 266 patients on the same day of surgery with an 80.5% success rate [17]. Although preoperative functional parameters were determined, postoperative IPSS, Qmax or PVR were not. In their prospective study, Carmignani et al. discharged 53 patients who underwent ThuVEP on the same day after surgery [15]. They reported functional parameters Qmax (9.3 ± 3.8 vs. 19.6 mL/s; *p* < 0.001) and IPSS (16 ± 3 vs. 6.5 ± 2) showing significant improvement on postoperative day 30.

#### Studies comparing SDD and non-SDD patient groups

In their prospective study, Cynk et al. performed HoLEP in a 184-patient cohort with the plan to discharge 114 patients on the same day at the postoperative 4th hour [16]. Of those, 90 (80%) could be discharged on the same day after successful surgery. Although they provided no data on pre- and postoperative IPSS, no statistical difference was observed comparing the two groups in terms of postoperative urinary flow rates and PVR volumes when day-case surgery (DCS) and non-DCS patient subgroups. Lwin et al. compared 199 same-day surgery (SDS) and 178 non-SDS patients who underwent HoLEP [18]. There was no significant difference between groups at their one-year follow-up in terms of Qmax, IPSS and PVR (9 ± 12 cm/s vs. 9 ± 10 cm/s for Qmax; *p* = 0.99, 11 ± 9 vs. 10 ± 9 for IPSS; *p* = 0.28 and 136 ± 216 vs. 117 ± 191 mL for PVR, *p* = 0.37).

Garden et al. performed a case–control matching of patients undergoing TURP, GreenLight photovaporisation (GL-PVP) and HoLEP; they compared the complications of patients with same-day discharge (SDD) to those of patients discharged at the standard length of hospital stay (SLD) [26]. However, they assessed no functional perioperative parameters. Another study by Assmus et al. compared patients who underwent urological/non-urological operations (e.g. urinary tract stone surgery, TURBT, bladder stone surgery, hydrocelectomy, partial cystectomy/diverticulectomy, etc., and/or general surgery operations) concurrently with HoLEP before and after transitioning a same-day discharge pathway facilitated by Moses 2. 0 Benign Prostatic Hyperplasia Mode [25]. Fifty-eight (62.4%) patients were discharged on the same day. In the whole cohort, their entire cohort’s postoperative AUASS improved compared to preoperative AUASS (21.7 vs 7.1; *p* < 0.001).

In their retrospective study including 473 patients undergoing HoLEP, Agarwal et al. divided patients into three groups as “planned inpatient admission” (PIA) (*n* = 266), “successful same-day discharge” (SDD) (*n* = 181) or “unplanned admission” (UA) (*n* = 26) [13]. They found postoperative AUASS values improved in SDD, PIA and UA groups (23.0 vs. 7.0, 20.0 vs. 6.0, and 19.5 vs. 5.0, respectively), but no difference between groups in terms of postoperative Qmax values (13.8 vs. 14.5 vs. 11.1, respectively; *p* = 0.7799).

### Perioperative complications, readmission and need for re-treatment

Table 5 (Online Resource 3) illustrates the success, readmission and complication rates of the studies we reviewed. In the Agarwal et al. study, eight patients complained of dysuria that resolved after a while at a median follow-up of 16 weeks (IQR 13–28.5), while 6 patients complained of transient urinary stress incontinence [24]. According to the Assmus et al. study, three patients (5.5%) from their whole cohort were readmitted postoperatively [14]. Of them, two patients were admitted to the emergency ward because of urinary tract infection (Clavien grade II) and urosepsis (Clavien grade IVa). Complications were observed in 13/55 (23.6%) patients among their entire patient group. Larner et al. reported that minor complications (phimosis, voiding failure, urethral catheter obstruction, left side pain and stenosis requiring bladder neck incision at week 6) occurred in five patients and three patients (7.89%) were readmitted after same-day discharge [27]. It is noted that patients aged > 75 years, on anticoagulation, with comorbidities and those with an ASA > 2 were not included in that study.

In another Comat et al. study, the overall complication rate was 36.7% and Clavien III complication rate only 3.3% [23]. The reasons for prolonged hospitalisation were gross haematuria requiring bladder irrigation (*n* = 13, 14.4%), dizziness due to tramadol administration (*n* = 1, 1.1%) and late admission to the operating room (*n* = 1, 1.1%). One patient underwent endoscopic surgical bladder clot removal under general anaesthesia (Clavien IIIb) six hours after HoLEP because of bladder clot retention. Three patients (3.3%) with severe haematuria requiring prolonged bladder irrigation, receiving erythrocyte suspension transfusion on postoperative day one. They reported that a low ASA score (*p* = 0.02) and advanced age (*p* = 0.04) were key risk factors for day-case surgery failure.

Lee et al. reported that four (5.5%) patients discharged on the same day were readmitted with deep vein thrombosis (*n* = 1), haematuria (*n* = 1), urinary tract infection (*n* = 1) and groin pain of unknown cause (*n* = 1) within 28 days (≤ Clavien grade II) [29]. Importantly, the study showed that performing the operation in the morning (OR 6.124, 95% CI 2.526–14.845, *P* < 0.001) and enucleation weight ≤ 40 g (OR 3.097, 95% CI 1.619–5.924, *P* = 0.001) were significant parameters affecting the success of same-day discharge.

Abdul-Muhsin et al. reported their experience on same-day HoLEP in a patient cohort including 179 patients of whom 47 were eligible for same-day discharge [12]. Readmission and non-readmission groups were compared. They found that the readmission group had a higher urinary tract infection (UTI) history (80% vs 26.2%, *p* = 0.0304) among patients discharged on the same day of surgery. Six complications developed in four patients who had been successfully sent home on the same day of surgery: these were Clavien grade II (epididymo-orchitis and UTI) and Clavien IIIa (fossa navicularis stenosis requiring dilatation in the office and clot retention requiring catheterisation and irrigation after initiating anticoagulation).

Klein et al. reported acute urinary retention (*n* = 14, 5.2%) and urinary infection (*n* = 6, 2.2%) as the reasons for readmission within 48 h after surgery (*n* = 18, 6.8%), namely, Clavien grade I–II complications [17]. Clavien III complications were observed in two patients (bladder clot removal on the first post-op day and reoperation to perform the morcellation phase, which was delayed because of a malfunctioning intraoperative morcellator). The only risk factor significantly associated with day-case surgery failure proved to be a prostate volume > 90 ml (OR = 2.041 *p* = 0.047). In the Carmignani et al. study, no patient required readmission, emergency admission or a blood transfusion. There were irritation symptoms in 8 patients that were resolved at week one [15]. Lee et al. reported a readmission rate of 6.4% in their entire cohort; the most common reason for admission was haematuria (30%). The same-day discharge rate in the m-HoLEP group was 87.9% and univariate and multivariate analyses showed that m-HoLEP was associated with same-day discharge [28].

### Studies comparing SDD and non-SDD patient groups

In their retrospective study, Riveros et al. analysed predictive factors for readmission within 30 days after HoLEP [30]. 2656 (76.1%) of 3489 patients were discharged within 24 h postoperatively. Readmitted patients were elderly, with preoperative anaemia, chronic renal disease, bleeding disorders and an ASA score ≥ 3. The most common causes of readmission were haematuria (27.22%) and UTI (6.33%). The complication rate of SDD patients was 4.7%. They found no significant difference in complication and readmission rates between their SDD and non-SDD patient groups (4.7% vs 4.0%, *P* = 0.004 and 3.7% vs. 4.8%, *p* = 0.2; respectively).

In the study by Cynk et al. that excluded patients with an ASA > 3, two patients had to be readmitted (2.2%) [16]. The reasons for prolonged hospital stay were haematuria (*n* = 9), nausea–vomiting (*n* = 5), high blood pressure (*n* = 2), allergic reaction (*n* = 1), sepsis-related confusion (*n* = 1), late operation (*n* = 2) and lack of a qualified urology nurse (*n* = 1). The morcellator malfunctioned in two patients during surgery, and re-do surgery was required the next day. Lwin et al. observed 27 (13.6%) and 36 (20.2%) complications in SDS and non-SDS groups, respectively (*p* = 0.14) [18]. Their SDS group’s complications were urinary retention (*n* = 9, 4.5%), urinary tract infection (UTI) (*n* = 10, 5.0%) and gross haematuria (*n* = 5, 2.5%) (Clavien grade I or II). They observed no statistically significant difference between their two groups in terms of postoperative complications. Their SDS cohort had a 2.5%. readmission rate: There was also no significant group difference in the 30-day readmission rate (%2.5 vs %4.5, *p* = 0.29).

Garden et al. compared the postoperative 30-day complication rates of TURP, GL-PVP and HoLEP procedures [26], finding no significant difference between SDD and SLD groups in terms of postoperative Clavien I/II and IV complications in HoLEP patients (3.64% vs. 3.86%; *p* = 0.802 vs. 0.34% vs. 0.8%; *p* = 0.205). In their study also investigating temporal discharge trends, they reported that the SDD rate dropped among GL-PVP patients and remained stable among TURP patients, but rising among HoLEP patients. The postoperative 90-day readmission rate was 7.9% in another study by Assmus et al. [25]. Clavien–Dindo complications ≥ IIIb did not differ significantly between their SDS and non-SDS groups (3.2% vs. 6.3%; *p* = 0.49). Similarly, another study by Agarwal et al. determining the SDD success rate in a cohort including 473 patients found no significant difference among PIA, successful SDD or UA groups in terms of Clavien ≥ III complications and complications within 90 days postoperatively (4.9% vs. 2.3% vs. 3.8%; *p* = 0.303 and 15.5% vs. 19.1% vs. 26.9%; *p* = 0.30, respectively) [13]. The causes of UA were haematuria requiring continuous bladder irrigation (*n* = 15; 58%), medical complication (n = 6; 23%), bladder injury during morcellation (n = 1; 4%) and inconclusively documented causes of UA (*n* = 4; %15).

Table 6 (Online Resource 3) presents pooled data from the prospective studies in the present review: the pooled postoperative IPSS and Qmax values of 5 prospective studies were 6.13 ± 2.84 (5.66–6.59) and 21.79 ± 13.75 (19.54–24.05) ml/s, respectively. In addition, the success rates of prospective studies in terms of day-case surgery were 78.2% and readmission rates were 8.8%.

## Discussion

Since laser EEP surgery has gained popularity all over the world in the last 20 years, urologists have much more experience with this surgery. Increasing surgical experience and good surgical outcomes have raised the question as to whether laser EEP can be done as an outpatient procedure [12, 16, 27]. Although day-case surgery raises patient safety concerns, it has obvious advantages. Briefer hospitalisations would probably result in fewer nosocomial infections [31], patients would be vulnerable to fewer complications, and therefore, costs would be indirectly lowered; thus, day-case surgery would be especially beneficial in economic terms. Moreover, it would enable a significant reduction in postoperative human resources. To the best of our knowledge, our study is the first review in the literature focussing on laser EEP taking the perspective of day-case surgery. In the studies we reviewed, improved functional parameters were also reported as expected.

In the present review we observed a day-case surgery success rate varying between 35.3 and 100%; the success rate of day-case surgery was 78.2%. Readmission rates ranged between 0 and 17.8% among the prospective studies, while in prospective studies it averaged 8.8%. We observed that haematuria was the most frequent reason for readmission. Satisfactory haemostasis is obviously essential in patients undergoing day-case surgery. Furthermore, postoperative urine colour monitoring and adequate irrigation should be carried out to avoid bleeding complications. Several studies within our review specifically examined GL-PVP or ThuVEP [15, 26]. Given the premise that vaporisation may lead to decreased bleeding and postoperative haematuria, it could be conjectured that this method possesses certain advantages over laser anatomical endoscopic enucleation of the prostate. Nevertheless, it is imperative to highlight that the comparative data garnered from the included studies is insufficient to conclusively affirm this hypothesis, particularly in relation to SDD.

The complication rates in the studies we reviewed ranged from 0% to 36.7%. The vast majority of complications were Clavien–Dindo I–II, reported at rates between 3.64 and 21.8%. Clavien ≥ III complications were between 0.34 and 10.7%. In studies comparing SDD to non-SDD groups, complication rates covering Clavien III and higher did not differ significantly between groups [18, 25, 26]. Clavien IV complications proved to be extremely seldom with a rate varying between 0 and 1.8%. One of the most important reasons for the low incidence of serious complications is that laser EEP surgery has become so widespread and that urologists’ experience with this procedure has increased over the years. Klein et al. observed that the SDD success rate has risen from 70 to 87% over the last years [17]. Moreover, laser EEP as a day-case intervention is now being perceived as a viable alternative. In their survey, Guo et al. queried intraoperative and postoperative surgical teams performing SDD after HoLEP [32] and 96% of the health professionals participating in their study agreed that SDD is safe following HoLEP.

Another possible reason for the low incidence of complications in SDD patients may be the fact that patients who underwent surgery on the same day were carefully selected patient groups. As we know, the general patient selection criteria for laser EEP are broad. Most patients with LUTS resulting from BPE can undergo laser EEP, regardless of prostate size, regardless of previous operations, urinary retention, non-neurogenic impaired bladder contractility, when re-treatment is required, and in patients on anticoagulation [33]. Laser EEP may also be preferable in patients with prostate cancer and obstructive symptoms, in those requiring re-treatment for benign prostatic hyperplasia or in patients needing concomitant surgery for other pathologies such as bladder stones, enabling very good functional results and low complication rates [34]. However, there is no consensus in the literature on which patients are best suited to form patient groups for specifically day-case surgery. Nevertheless, some patient characteristics such as age, ASA status, prostate volume and anticoagulant use can be taken into account when assessing the success of DCS. In a retrospective French study, age (*P* = 0.019), ASA score > 2 (*P* = 0.0019), larger prostate volume (*P* = 0.011) and anticoagulant use (*P* < 0.0001) were associated with the risk of complications of DCS for HoLEP [35]. Kosiba et al. showed that prostate volume (OR 1.01) and a high ASA score (OR 2.29) were significant predictors of major complications after HoLEP surgery [36]. In the present study, some of the studies we reviewed excluded patients with an ASA > 2 and/or elderly patients [27]. Anticoagulant use was an exclusion criterion in some studies [14, 23, 25, 27], while in others, ASA > 3 patients did not qualify for same-day surgery [12, 16]. Even the distance between the patient's home and the emergency ward was a reason for exclusion in one study [23]. The fact that patients with high ASA scores, elderly patients and/or those on anticoagulants are usually excluded may be interpreted as being associated with low rates of postoperative complications in DCS of laser EEP. Mean prostate size, duration of indwelling catheter and even the postoperative discharge time of the patient in defining day-case surgery differed among studies. Although the studied we reviewed had no homogeneous patient selection criteria, the low perioperative complication rates and generally low Clavien–Dindo I–II complication rates they report are encouraging and seem to indicate a promising future for the day-case performance of laser EEP. In addition, the fact that same-day discharge is possible even after some urological and general surgical interventions are done concomitant with laser EEP is a noteworthy point when considering the range of SDD suitability after laser EEP [25]. However, we emphasise that there is a need for consensus on specific patient selection criteria for DCS.

Predictive factors for the success of day-case surgery and readmission were identified in some studies we reviewed. Prostate size stands out as one of these factors. It is unclear what the maximum prostate size would be to rule out SDD following laser EEP. Lee et al. showed that morning surgery and a prostate size less than 40 g are factors affecting success [29]. Klein et al. reported that prostate size > 90 ml is the most important factor for failed SDD after laser EEP [17]. Assmus et al. demonstrated the success of day-case surgery in prostates > 175 cc [14]. The mean prostate size in the studies in our review ranged from 35 ± 11.4 to 229 ml. We therefore conclude that medium-to-large prostates are SDD suitable after laser EEP. Riveros et al. investigated independent predictors of readmission after laser EEP: a bleeding disorder (OR 2.89, 95% CI 1.63e5.11, *P* < 0.001), ASA score ≥ 3 (OR 1.80; 95% CI 1.21–2.70; *P* = 0.004) and high frailty burden (OR 1.67; 95% CI 1.03–2.71; *P* = 0.038) [30]. Another interesting study found that a low ASA score and advanced age were significant risk factors for SDD failure [23]. These authors explained that most of their study patients with an ASA score 2 could not be discharged on the same day because they were on antiplatelet therapy and needed more attention to ensure haemostasis. On the other hand, Abdul-Muhsin et al. reported no significant predictor for early discharge or readmission after laser EEP in their study [12]. In terms of the studies we reviewed, factors such as age, ASA score, prostate size and bleeding disorders/anticoagulant use seem to be the main factors predicting SDD success.

Apart from prostate size and ASA scores, we must keep in mind that the surgeon's experience and the hospital’s case volume are general success factors, as in other types of surgeries. In a multicentre study by Khene et al. involving 6 surgeons and 992 patients who had undergone GreenLight laser EEP, surgical experience was associated with shorter enucleation and morcellation time (*P* < 0.001), lower intraoperative complication rates (*P* < 0.001) and improvement in IPSS at 3 months postoperatively (*P* = 0.004) [37]. Similarly, in another multicentre study involving 5 centres with 39 surgeons and more than 1000 HoLEP cases, more surgical experience proved to have a positive effect on operation and enucleation times and contributed to less postoperative urinary incontinence [38]. We can therefore conclude that an experienced surgeon and a centre’s case volume are crucial for patient safety during the perioperative period for DCS success following laser EEP. Another important factor for successful DCS is having a skilled and well-trained postoperative care team [12], which is vital for a patient's SDD decision. After the patient leaves the operating room, this team monitors the vital parameters, urine colour, severity of haematuria, the surgeon in charge is informed and the decision whether the patient will be discharged on the same day is made according to the postoperative care team’s follow-up. The postoperative care team needs to be specially trained and informed about day-case surgery. Ideally, the day-surgery nursing staff should not change frequently, and we would recommend that operation lists be organised according to the days when these trained nurses are on duty.

With regard to nosocomial infections, it is worth adding a few comments on why SDS may be advantageous. In the studies we reviewed, there are no data showing a relationship between length of hospital stay and the risk of nosocomial infection. However, the literature has shown an increase in nosocomial infections with prolonged hospitalisation [39]. Furthermore, in the European Association of Urology 2023 Guidelines on Urological Infections, it is clearly stated that one of the most effective methods to prevent nosocomial infections/urosepsis is to reduce the length of hospital stay [31]. Since same-day discharge also means a very short hospital stay, we believe it would not be wrong to assume that it is advantageous in terms of preventing nosocomial infections.

In conclusion, several aspects related to DCS and its financial implications merit further discussion. Existing literature reveals that HoLEP and GreenLight vaporisation demonstrate financial advantages over TURP and OP due to reduced hospital stays and morbidity [40–42]. Ostensibly, due to shorter hospital stays, decreased nosocomial infection rates and consequent potentially lower treatment costs, DCS could present fiscal advantages. A preponderance of studies encompassed in our review implies that SDS could be economically beneficial. Yet, only one study included in this review conducted a cost analysis, comparing m-HoLEP with standard HoLEP. It is essential to underscore that this study did not conduct a cost analysis comparing patients discharged on the same day versus those not discharged on the same day. Hence, although we can hypothesise about potential cost benefits associated with SDS, there remains insufficient evidence to substantiate these projections, constituting a significant limitation of this study. Further, healthcare policies, systems, surgical costs and reimbursement policies exhibit considerable variability across different countries. Hence, considerations around daily surgical expenditures, postoperative patient readmissions and associated reimbursements will underpin potential cost savings. We maintain that to determine conclusively the cost saving implications of DCS, varying reimbursement policies and hospital readmission costs must be thoroughly evaluated.

## Study limitations

This systematic review is also not without limitations. Firstly, most of the studies in our review are retrospective. Different inclusion, exclusion, same-day discharge criteria and postoperative readmission times in the studies are other important limitations. As mentioned, one of the most important limitations is the lack of a detailed cost analysis of day surgery, which is one of the most important considerations as to whether SDS is worthwhile for laser endoscopic enucleation of the prostate. In addition, factors such as different numbers of patients included, different prostate sizes, anatomical features, operation and medication histories of the patients, various catheter removal times and different experience levels of the surgeons in the studies led to heterogeneous data, thus introducing potential biases. Furthermore, long-term outcomes such as the duration of functional recovery, complications and readmission rates beyond the postoperative period were not assessed in all the studies included in our review.

## Conclusions

Laser EEP is feasible and promising as a day-case surgery enabling improved functional parameters and low perioperative complication and readmission rates in certain patients. However, the question as to which patient group is suitable for daily surgery remains unanswered. We believe that prospective studies enrolling larger patient cohorts should be conducted to determine the exact patient selection criteria for laser EEP as a day-case surgery, and thus the suitability and feasibility of laser EEP as an outpatient procedure should be more clearly demonstrated.

### Supplementary Information

Below is the link to the electronic supplementary material.

Supplementary file1 (PDF 264 KB)

Supplementary file2 Figure 1: Flow diagram of the study selection process (PDF 153 KB)

Supplementary file3 (PDF 157 KB)

## Data Availability

The raw data are available with the corresponding author and can be provided on request.
