# Supplementary material for: Is day-case surgery feasible for laser endoscopic enucleation of the prostate? A systematic review
Source: World J Urol. 2023 Sep 10;41(11):2949–58. doi: 10.1007/s00345-023-04594-7 (PMC10632304; doi:10.1007/s00345-023-04594-7)
Supplement: Supplementary file 1 — Supplementary file1 (PDF 264 KB) [file 345_2023_4594_MOESM1_ESM.pdf]

Table 4: Intraoperative and postoperative data of the patients

Author and year		Enucleated weight (g)	ET (min)*	MT (min)*	EE (g/min)*	ME (g/min)*	OT (min)*	Post-op IPSS *	Post-op Qmax (ml/s)*	Post-op QoL*	Post-op PVR *	Post-op time duration to discharge
Assmus et al., 2021a	SDS	78 (25 to 119)	38 (28-55)	8 (4-15)	1.65 (0.90-2.63)	9.36 (7.08-10.55)	99 (80-139)	7.1	15	N/A	N/A	3:49 (2:10–23:00)
	Non-SDS	66 (48 to 88)	44 (28 -56)	8 (6-12)	1.41 (0.95-2.40)	6.50 (5.51-9.07)	83 (74-92)					23:11 (20:18–26:49) (hh:mm)
Agarwal et al., 2020		52.5 (33-81)	39.5 (30.5-53)	5 (4-12)	N/A	N/A	N/A	5 (2-5) [a]	N/A	1 (0-2)	16 (8-37)	2.6 (2.1-2.9) (hours)
Garden et al., 2022		N/A	N/A	N/A	N/A	N/A	N/A	N/A	N/A	N/A	N/A	N/A
Lee et al., 2021		N/A	42.2 (17.2)	10.9 (12.0)	N/A	N/A	74.5 (35.4)	N/A	N/A	N/A	N/A	N/A
Carmignani et al., 2015		27.5±10 (15–45)	N/A	N/A	N/A	N/A	71±32 (35–105)	6.5±2 (4–8) (30.day)	19.6 mL/s (8–51.8) (30.day)	N/A	N/A	2 hours after catheter removal
Klein et.al, 2020	Success	43.2 (±30.3)	N/A	N/A	N/A	N/A	62.5 (±27.6)	N/A	N/A	N/A	N/A	<12 hours
	Failure	54.9 (±36.5)					72.0 (±29.1)					
Cynk et al., 2015	DCS	22.6 (0.5-125.5)	N/A	N/A	N/A	N/A	N/A	N/A	20.1 (7-61.3)	N/A	N/A	4 hours
	Non-DCS		N/A	N/A	N/A	N/A	N/A	N/A		N/A	N/A	N/A
Abdul-Muhsin et al., 2020		N/A	35.5 (16-126)	N/A	N/A	N/A	98 (39-190)	N/A	N/A	N/A	N/A	N/A
Larner et al., 2003		7±4.8	N/A	N/A	N/A	N/A	60±22.1	6.6±6.8(3rd month)	18.4±8(3rd month)	1.2±1(3rd month)	N/A	302±86 (min)
Comat et al. 2017		40 (2-53)	N/A	N/A	N/A	N/A	70 (10-180)	5.04 (0–18) (1st month)	24.9 (6.2-74) (1st month)	N/A	N/A	<12 hours
Assmus et al., 2021b		165.1 (45-385)	72.5 (23-133)	23.3 (2-113)	N/A	N/A	121.6 (37-243)	6.7 (2-12) (3th month)	20.4 (10.9–29.9) (3th month)	1.3 (0-2) (3th month)	25 (0-109)	2.7 hours
Lwin et al., 2020	SDS	53±41	N/A	N/A	N/A	N/A	110±47	6±5 (12th month)	15±11 cm/s (12th month)	N/A	56± 88 (12th month)	3.9±1.8(hours)
	Non-SDS	66±47	N/A	N/A	N/A	N/A	132±67	6±6 (12th month)	14±9 (12th month)	N/A	62±98 (12th month)	30.5±15.8

Lee et al., 2018	SDS	23 (1-120)	N/A	N/A	N/A	N/A	61 (21-163)	N/A	N/A	N/A	N/A	<23 hours
	Non-SDS	50 (1-295)	N/A	N/A	N/A	N/A	86 (22-284)	N/A	N/A	N/A	N/A	N/A
Agarwal et al., 2022	SDS	49 (21-86)	39 (29-52.5)	5 (2-10)	N/A	N/A	N/A	7 (4-12) [a] (3rd month)	13.8(9.1-21.3) (3rd month)	N/A	28(16-72) (3rd month)	N/A
	PIA	56 (25.5-92)	45 (33-58)	7 (3-13)	N/A	N/A	N/A	6 (3-11) [a] (3rd month)	14.5 (9.7-21) (3rd month)	N/A	30.5 (0-67) (3rd month)	N/A
	UA	72 (43-96)	39.5 (33-58)	8 (3-13)	N/A	N/A	N/A	5 (4-13) [a] (3rd month)	11.1 (8.7-20.8) (3rd month)	N/A	15.5 (12-47) (3rd month)	N/A
Riveros et al., 2022	SDD	N/A	N/A	N/A	N/A	N/A	95 (61-132)	N/A	N/A	N/A	N/A	<24 hours
	Non-SDD											>24 hours

* Mean± SD or median (range) (IQR)
[a] AUASS: American Urological Association Symptom Score

Abbreviations: SDS: same day surgery; DCS: day-case surgery; PIA: planned inpatient admission; UA: unplanned admission; N/A: not applicable; ET: Enucleation time; MT: Morcellation time; EE: Enucleation efficiency; ME: Morcellation efficiency; OT: Operation time; ; IPSS:International Prostate Symptom Score; Qmax: maximum urinary flow rate; QoL: quality of life; PVR: post-voiding residual volume

Table 5: Success, readmissions rates and complications according to Clavien-Dindo classification

Author and year		Success rate (%)	Readmission, n (%)	Reason for readmission	Readmission management	Complications<br>Clavien-Dindo classification, n (%)				
						I	II	III	IV	V
Assmus et al., 2021a		N/A	7.9	N/A	N/A	N/A	N/A	3 (3.2)	0	0
Agarwal et al., 2020		Not reported	0	0	0	N/A	N/A	N/A	N/A	N/A
Garden et al., 2022		N/A	17 (1.92)	N/A	N/A	32 (3.64)		0	3 (0.34)	0
Lee et al., 2021		N/A	20 (6.4)	Catheter issue<br>Retention<br>Hematuria<br>Urinary tract infection<br>Gastrointestinal<br>Respiratory<br>Musculoskeletal<br>Neurological	N/A	N/A	N/A	N/A	N/A	N/A
Carmignani et al., 2015		100	0	N/A	N/A	N/A	N/A	N/A	N/A	N/A
Klein et.al, 2020		80.5	7.4	Acute urinary retention, urinary infection	N/A	20 (7.4)		2 (0.75)	N/A	N/A
Cynk et al., 2015	DCS	80	2.6	hematuria	observation	N/A	N/A	N/A	N/A	N/A
	Non-DCS	N/A				N/A	N/A	N/A	N/A	N/A
Abdul-Muhsin et al., 2020		59.5	17.8	hematuria	N/A	0	3 (6.4)	3 (6.4)	0	0
Larner et al., 2002		Not reported	10	Paraphimosis, failed trial of voiding, blocked catheter, worsening stream	Re-catheter, suprapubic catheter, bladder neck incision	N/A	N/A	N/A	N/A	N/A
Comat et al. 2017		80	11 (12.2)	AUR	N/A	13 (14.4) (in 90 days)	20 (22.2) (in 90 days)	3 (33.3) (in 90 days)	0	0
Assmus et al., 2021b		84	3 (5.5)	urinary tract infection, acute kidney injury, hematuria/clots and fever	Short-term dialysis, antibiotic therapy	5 (9.1)	7 (12.7)	0	1 (1.81)	0
Lwin et al., 2020	SDS	N/A	5 (2.5)	AUR, urinary tract infection<br>Gross hematuria	N/A	14 (7)	13 (6.5)	0	0	0
	Non-SDS	N/A	8 (4.5)	AUR, urinary tract infection<br>Gross hematuria	N/A	17 (9.6)	10 (5.6)	5 (2.8)	4 (2.2)	0
Lee et al., 2018		35.3	4 (5.5)	deep vein thrombosis, haematuria, urinary tract infec- tion and groinpain of unknown cause	Conservatively, antibiotic, anticoagulation	N/A	N/A	N/A		
Agarwal et al., 2022	SDS	87.4	22 (12) in 90 days	N/A	N/A	N/A	N/A	9 (4.9)		
	PIA	N/A	N/A	N/A	N/A	N/A	N/A	6 (2.3)		
	UA	N/A	N/A	N/A	N/A	N/A	N/A	1 (3.8)		
Riveros et al., 2022		N/A	31 (3.7)	Hematuria<br>urinary tract infection	N/A	N/A	N/A	N/A	N/A	N/A

* Mean±SD or median (range) (IQR)

Abbreviations: SDS: same day surgery; DCS: day-case surgery; PIA: planned inpatient admission; UA: unplanned admission; N/A: not applicable; AUR: acute urinary retention

Table 6: Pooled postoperative functional outcomes, success and readmission rates of prospective studies						
	Patient number	Postop IPSS*	Post-op Qmax (ml/s)*	Post-op PVR *	Success rate (%)	Readmission rate, n (%)
Carmignani et al.	53	6.5±2 (4–8)	19.6 (8–51.8)	N/A	100	0
Klein et al.	214	N/A	N/A	N/A	80.5	7.40
Cynk et al.	111	N/A	N/A	N/A	80	2.60
Abdul-Muhsin et al.	47	N/A	N/A	N/A	59.5	17.80
Comat et al.	90	5.04 (0–18)	24.9 (6.2-74)	N/A	80	11 (12.2)
		6.13 ± 2.84 (5.66- 6.59) ¶	21.79 ± 13.75 (19.54- 24.05) ¶	N/A ¶	78.2 (74-81) ¶	8.8 (6.6-12) ¶

*Mean±SD or median (range) (IQR)

¶ Pooled data

IPSS:International Prostate Symptom Score; Qmax: maximum urinary flow rate; PVR: post-voiding residual volume
