# Supplementary material for: Is day-case surgery feasible for laser endoscopic enucleation of the prostate? A systematic review
Source: World J Urol. 2023 Sep 10;41(11):2949–58. doi: 10.1007/s00345-023-04594-7 (PMC10632304; doi:10.1007/s00345-023-04594-7)
Supplement: Supplementary file 2 — Supplementary file2 Figure 1: Flow diagram of the study selection process (PDF 153 KB) [file 345_2023_4594_MOESM2_ESM.pdf]

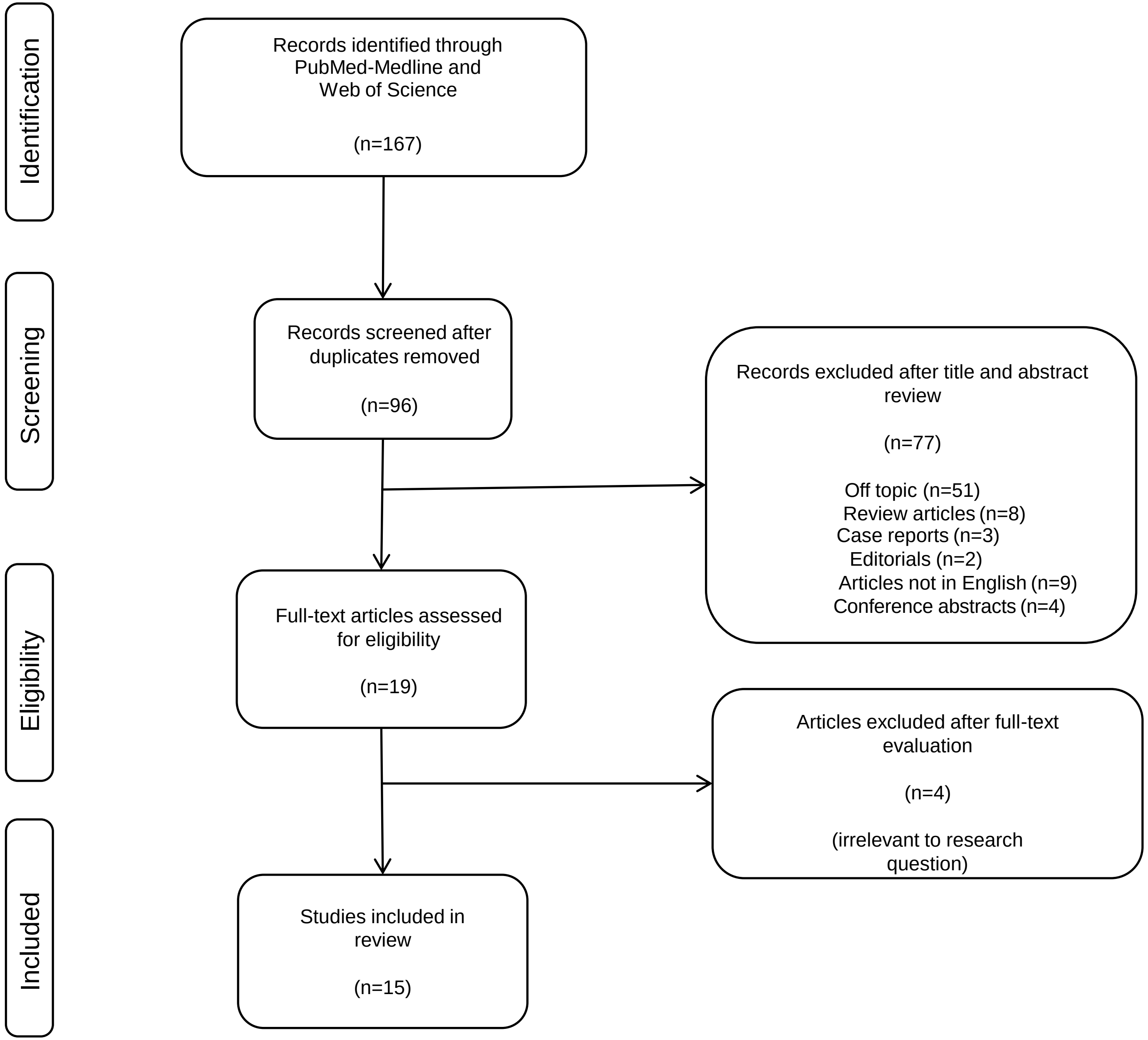
Identification
Records identified through PubMed-Medline and Web of Science
(n=167)
Screening
Records screened after duplicates removed
(n=96)
Records excluded after title and abstract review
(n=77)
Off topic (n=51)
Review articles (n=8)
Case reports (n=3)
Editorials (n=2)
Articles not in English (n=9)
Conference abstracts (n=4)
Eligibility
Full-text articles assessed for eligibility
(n=19)
Articles excluded after full-text evaluation
(n=4)
(irrelevant to research question)
Included
Studies included in review
(n=15)

Table 1: Quality assessments of the studies according to NIH Quality Assessment Tool													
Studies	Quality assessment criteria												Quality
	1	2	3	4	5	6	7	8	9	10	11	12	
Assmus et al., 2021a	+	+	+	+	-	+	+	-	+	+	-	+	75% good
Agarwal et al., 2020	+	+	+	+	-	+	+	-	-	-	-	+	58% poor
Garden et al., 2022	+	+	+	+	+	+	-	-	N/A	-	-	-	50% poor
Lee et al., 2021	+	+	+	+	+	+	+	-	N/A	-	-	+	66% adequat e/fair
Carmignani et al., 2015	+	+	+	+	+	+	+	-	+	+	-	+	83% strong
Klein et.al, 2020	+	+	+	+	+	+	+	-	N/A	-	-	+	66% adequat e/fair
Cynk et al., 2015	+	+	+	+	+	+	+	-	-	-	-	+	66% adequat e/fair
Abdul-Muhsin et al., 2020	+	-	+	+	+	+	+	-	N/A	N/A	N/A	+	58% poor
Larner et al., 2003	+	+	+	+	-	+	+	-	+	+	-	+	75% good
Comat et al., 2017	+	+	+	+	+	+	+	-	+	+	-	+	83% strong
Assmus et al., 2021b	+	+	+	-	+	+	+	-	N/A	-	-	+	58% poor
Lwin et al., 2020	+	+	+	+	+	+	+	-	+	+	-	+	83% strong
Agarwal et al., 2022	+	+	+	+	+	+	+	-	+	+	-	+	83% strong
Riveros et al., 2022	+	+	+	-	+	+	+	-	N/A	-	-	-	50% poor
Lee et al., 2018	+	+	+	+	+	+	+	-	N/A	-	-	-	58% poor

Table 2: Inclusion and exclusion criteria for SDD in the studies		
	Inclusion criteria	Exclusion criteria
Assmus et al., 2021a	• patients who underwent HoLEP with concurrent surgeries	• lack of a caregiver and significant comorbidities (including ASA score ≥4)<br>• patients on anticoagulation or dual antiplatelet therapy
Assmus et al., 2021b	• large gland prostates (≥175 cc)	• lack of a caregiver<br>• significant comorbidities (including ASA score ≥4, inability to hold therapeutic anticoagulation, or dual anti- platelet therapy)
Agarwal et al., 2020	• same day catheter removal and SDD after HoLEP	• patients who were not offered same day catheter removal were those with prostate volumes >250 ml
Garden et al., 2022	• patients who underwent HoLEP	• ASA score ≥4 or unknown<br>• non-elective or emergency surgery<br>• discharge to a non-home location<br>• possession of ≥1 major comorbidities (wound infection, requiring blood transfusion within ≤72 hours before surgery, bleeding disorder, acute renal failure, end-stage renal disease, ventilator-dependence, preoperative sepsis, or disseminated cancer<br>• missing data<br>• prolonged length of stay
Lee et al., 2021	• patients who underwent HoLEP	• not reported
Carmignani et al., 2015	• patients who underwent ThuVEP as a 1-day surgery.	• prostate carcinoma<br>• concomitant urethral strictures<br>• bladder tumors<br>• history of urethral or prostatic surgery.
Klein et.al, 2020	• all consecutive day-case HoLEP cases	• high risk for a complication after general anesthesia (unstable medical conditions)<br>• living too far from a hospital with an emergency unit (> 150 km)<br>• being alone at home the night after surgery
Cynk et al., 2015	• all patients with bladder outflow obstruction, for which the preferred management option was surgery	• ASA score >3<br>• >1 h return time to hospital<br>• Lack of adult supervision for first postoperative night<br>• Anticoagulant therapy which could not be stopped<br>• At discretion of anaesthetic consultant at pre-admission<br>• At discretion of consultant urological surgeon
Abdul-Muhsin et al., 2020	• same-day discharge following HoLEP	• Presence of prostate cancer<br>• ASA score > 3<br>• prostate volume > 200 cc<br>• age > 75 years<br>• lack of care giver<br>• the location of residence is outside the city limits<br>• enucleation time >1 h<br>• morcellation time >30 min,<br>• post-operative hemoglobin change >2 g/dL<br>• surgery end time after 1 pm
Larner et al., 2003	• patients who underwent HoLEP with a prostate volume of < 60 mL	• age >75 years<br>• ASA score ≥2<br>• significant comorbidities (undetailed)<br>• patients on anticoagulation<br>• with indwelling catheter

		• history of previous prostatic surgery<br>• Qmax >15 mL/s and IPSS <15
Comat et al. 2017	• patients experiencing LUTS due to BPH and who were candidates for HoLEP	• unfit medical condition (e.g. unstable cardiovascular disease, anticoagulant therapy)<br>• long distance from home to any emergency unit (>150 kilometres)<br>• general state unable for day-case surgery (e.g. difficulty walking, cognitive disorder)<br>• not being accompanied by an adult the night after surgery (due to legal issues in authors' country)
Lwin et al., 2020	• Patients underwent HoLEP<br>• living in the local metropolitan area with immediate access to an emergency room<br>• ECOG status 0-2	• ECOG status 3-5<br>• patients living outside the local metropolitan area<br>• patients unwilling to be sent home with an indwelling catheter<br>• If the patient was admitted to the hospital for observation and discharged within 24 hours, this was not classified as SDS
Lee et al., 2018	• consecutive patients undergoing HoLEP<br>• National Early Warning Score of 0<br>• mobilising<br>• tolerating oral intake, acceptable haematuria and freely draining catheter.	• patients who did not meet inclusion criteria<br>• patients who had prolonged or late anaesthetic recovery<br>• patients who declined day-case discharge
Agarwal et al., 2022	• patients undergoing HoLEP	• if social reasons prevented SDD<br>• patients who was felt they would medically benefit from postoperative observation/care
Riveros et al., 2022	• patients (≥18 years) who underwent HoLEP	• age >90 years<br>• ventilator-dependent at the time of surgery<br>• ASA score 5<br>• history of ascites<br>• acute renal failure<br>• sepsis prior to surgery<br>• unknown age, ASA score, height, weight, preoperative functional health status, preoperative hematocrit and serum creatinine, operative time, length of hospital stay, or discharge destination
Abbreviations: HoLEP: Holmium laser enucleation of the prostate; ThuVEP: Thulium vapoenucleation of the prostate; IPSS: International Prostate Symptom Score; Qmax: maximum urinary flow rate; ASA: American Society of Anesthesiologists; SDD: same-day discharge; SDS: same-day surgery; LUTS: lower urinary tract symptoms; BPH: benign prostatic hyperplasia;		
