# Supplementary material for: Is day-case surgery feasible for laser endoscopic enucleation of the prostate? A systematic review
Source: World J Urol. 2023 Sep 10;41(11):2949–58. doi: 10.1007/s00345-023-04594-7 (PMC10632304; doi:10.1007/s00345-023-04594-7)
Supplement: Supplementary file 3 — Supplementary file3 (PDF 157 KB) [file 345_2023_4594_MOESM3_ESM.pdf]

Table 3: Summary of demographics, baseline characteristics and perioperative data of studies

Author, year, study design	Procedure		Preop PV-TRUS or TAUS (ml)*	Nr. of patients	Age (years) *	PSA (ng/ ml) *	Pre-op IPSS *	Pre-op Qmax* (ml/s)	Pre-op PVR*	Pre-op QoL*	Previous prostate surgery, n (%)	CT (day)*	History of urinary retension, n (%)	Preop indwelling catheteter	Use of 5-ARI, n (%)	Use of AP/AC therapy, n (%)	Morbiditiy, n (%)	ASA score, n (%)
Assmus et al., 2021a, retrospective	HoLEP	SDS	124.5 (51.8-161.3)	93	71.7 (65.8-77.0)	6.93	21.7	6.67	N/A	N/A	N/A	18:34 (6:24–22:14)	40%	N/A	N/A	N/A	N/A	3
		Non-SDS	107.5 (79.8-129.6)	95	74.9 (68.7 - 79.6)	5.93						18:24 (16:08–21:17) (hour:min)	32%					3 (median)
Agarwal et al., 2020, retrospective	HoLEP		81 (53-115)	30	68.6 (61.7-73.3)	N/A	18 (13-29)[a]	6.4 (5.1-8.5) (n=8)	82 (30-132)	4 (3.5-5.5)	4 (13.3)	4.9 hours (3.5-6.0)	8 (27)	9 (30)	6 (29) (combination with alpha blocker)	6 (20)	N/A	1-2: 17 (57)<br>3: N/A<br>4: N/A
Garden et al., 2022, retrospective	HoLEP	SDD	N/A	880	Median age not reported	N/A	N/A	N/A	N/A	N/A	N/A	N/A	N/A	N/A	N/A	N/A	N/A	1-2: 471 (53.52)<br>3: 409 (46.48)
		SLD		880														1-2: 471 (53.52)<br>3: 409 (46.48)
Lee et al., 2021, retrospective	HoLEP	HoLEP	115.8 (90.4)	120	69.76 (8.44)	N/A	N/A	N/A	N/A	N/A	N/A	N/A	55 (45.8)	39 (45.9)	N/A	22 (36.1)	N/A	1: 3 (2.5)<br>2: 35 (29.2)<br>3: 78 (65.0)<br>4: 4 (36.4)
		m-HoLEP	114.8 (73.2)	120	71.04 (8.96)								64 (33.3)	46 (54.1)		39 (63.9)		1: 5 (2.6)<br>2: 84 (43.8)<br>3: 94 (49.0)<br>4: 7 (3.6)
Carmignani et al., 2015, prospective	ThuVEP		68.9±20.1	53	71.8±8.8	4.22 ±2.45	16±3	9.3±3.8	N/A	3.5±1	N/A	14.8 (hours)	N/A	5 (9.4)	N/A	7 (13.2)	N/A	N/A
Klein et.al, 2020, prospective	HoLEP	Success	77.4 (±41.9)	214	66.6 (±7.39)	5.04 (±5.16)	18.0 (±6.95)	8.77 (±4.13)	127 (±127)	N/A	N/A	N/A	N/A	17 (8.3%)	N/A	35 (16)	N/A	1,77 (±0,569)
		Failure	91.0 (±37.8)	52	67.9 (±8.34)	6.28 (±4.31)	18.4 (±5.84)	8.35 (±3.89)	142 (±123)					5 (10%)		4 (7.7)		1,61 (±0,568) (median±SD)

Cynk et al., 2015, prospective	HoLEP	DCS	N/A	111	74 (48-92)	N/A	N/A	8.65 (2.3-24.3)	N/A	N/A	N/A	N/A	107 (58)	77 (42)	N/A	0	N/A	1: 17 (15)<br>2: 75 (68)<br>3: 19 (17)<br>4: 0
		Non-DCS	N/A	68		N/A	N/A		N/A	N/A	N/A	N/A			N/A	10 (14.3)	N/A	1: 6 (9)<br>2: 36 (51)<br>3: 24 (34)<br>4: 2 (3)
Abdul-Muhsin et al., 2020, prospective	HoLEP		70.6 (20.0–165.0)	47	69.7 (56.9–83.0)	N/A	N/A	N/A	N/A	N/A	8 (17)	1 (1-6)	N/A	10 (21.3)	13 (27.7)	9 (19.1)	3 (1-9) (Charlson comorbidity index)	1: N/A<br>2: 31 (66)<br>3: 16 (34)<br>4: N/A
Larner et al., 2003, retrospective	HoLEP		35±11.4	38	64.5±6.4	N/A	23.8±7.3	6±3.3	N/A	4.3±1	None	2.6±1.5	N/A	None	N/A	None	None	1: 38 (100)
Comat et al. 2017, prospective	HoLEP	Day-case	65 (26-180)	90	65 (46-83)	3.27 (0.28-25)	20 (6-35)	7.4 (2-22)	100 (0-900)	N/A	N/A	24 (20-96) (hours)	N/A	5 (5.6)	N/A	8 (11.1)	N/A	1 and 2: 90 (100)
		Conventional hospitalisation	70 (20-257)	121	71 (49-92)	4.22 (0.32-39.1)	18 (4-33)	9.4 (2.2-30)	102.5 (0-1200)			N/A		N/A		32 (26)	N/A	>2: 31 (26)
Assmus et al., 2021, retrospective	HoLEP		229 (175-535)	45	73.8 (56-91)	8.58 (2.7-15.66)	22.3 (2-35)[a]	8.8 (2.7-19.5)	172 (37-600)	4.9 (2-6)	5 (9)	17 (hours)	43 (78.2)	32 (58.2)	N/A	8 (18)	None	2.6 (2-4) mean (range)
Lwin et al., 2020, retrospective	HoLEP	SDS	83±49	199	70 (49-89)	8±12	19 ±8	6±4	191±224	N/A	36 (16.5)	32 (1-337)	86 (43.2)	N/A	79 (39.7)	N/A	N/A	N/A
		Non-SDS	96±55	178	71 (55-94)	8±7	20±25	5±3	179±207	N/A	19 (10.7)	32 (1-361)	97 (54.5)	N/A	69 (38.8)	N/A	N/A	N/A
Lee et al., 2018, retrospective	HoLEP	SDS	N/A	74	68.9±8.1	N/A	N/A	N/A	N/A	N/A	N/A	N/A	37 (50)	25 (33.8)	N/A	N/A	N/A	1: 10 (13.5)<br>2: 52 (70.3)<br>3: 8 (10.8)<br>Not documented: 4 (5.4)
		Non-SDS	N/A	136	71.1±8.7	N/A	N/A	N/A	N/A	N/A	N/A	N/A	83 (61)	58 (42.6)	N/A	N/A	N/A	1: 20 (14.7)<br>2: 98 (72.1)<br>3: 18 (13.2)
Agarwal et al., 2022, retrospective	HoLEP	SDS	88 (60-121)	181	69 (8.9)	4.6 (2.1-8.8)	23 (16-27) [a]	N/A	90.5 (48-135)	N/A	16 (8.8)	N/A	N/A	53 (29.3)	55 (30.4)	29 (16.3)	N/A	3-4: 105 (58)

		PIA	87 (60-128)	266	72 (10.4)	5.8 (2.9-8.8)	20 (15-26) [a]	N/A	111 (54-200)	N/A	35 (13.2)	N/A	N/A	95 (36.1)	73 (27.8)	66 (25)	N/A	3-4: 197 (74.1)
		UA	82(50-119)	26	72 (8.1)	6.2 (3.9-10.3)	19.5 (10.5-24.5) [a]	N/A	51.5 (41-131.5)	N/A	3 (11.5)	N/A	N/A	7 (26.9)	7 (26.9)	6 (23.5)	N/A	3-4: 18 (69.2)
Riveros et al., 2022, retrospective	HoLEP	SDD	N/A	833	70.00 [65.00, 76.00]	N/A	N/A	N/A	N/A	N/A	N/A	N/A	N/A	N/A	N/A	N/A	N/A	1-2: 413 (49.6) 3-4: 420 (50.4)
		Non-SDD		2656	70.00 [64.00, 76.00]													1-2: 1410 (53.1) 3-4: 1,246 (46.9)

Abbreviations: HoLEP: Holmium laser enucleation of the prostate; ThuVEP: Thulium vapoenucleation of the prostate; PV: prostate volume; PSA: prostate spesific antigen; IPSS: International Prostate Symptom Score; Qmax: maximum urinary flow rate; QoL: quality of life; LOS: Length of hospital stay; OT: operation time; N/A: not applicable; CT: Catheterization Time; TRUS: transurethral ultrasonography, TAUS: transabdominal ultrasonography; AP/AC: antiplatelet/anticoagulation; 5-ARI: 5-alpha-reductase inhibitor; ASA: American Society of Anesthesiologists

*Mean±SD or median (range) (IQR)

[a] AUASS: American Urological Association Symptom Score
